# Emerging microfluidics-enabled platforms for osteoarthritis management: from benchtop to bedside

**DOI:** 10.7150/thno.62685

**Published:** 2022-01-01

**Authors:** Zhou Zou, Xiaohe Luo, Zhengkun Chen, Yu Shrike Zhang, Chunyi Wen

**Affiliations:** 1 Department of Biomedical Engineering, Faculty of Engineering, Southern University of Science and Technology, Shenzhen, Guangdong, China.; 2 Department of Biomedical Engineering, Faculty of Engineering, The Hong Kong Polytechnic University, Hong Kong, China.; 3 Currently at Department of Chemistry, University of Toronto, Toronto, Ontario M5S 3H6, Canada.; 4 Division of Engineering in Medicine, Department of Medicine, Brigham and Women's Hospital, Harvard Medical School, Cambridge, Massachusetts 02139, United States.; 5 Research Institute of Smart Ageing, The Hong Kong Polytechnic University, Hong Kong, China.

**Keywords:** Osteoarthritis, microfluidics, organ-on-a-chip, biosensor, intra-articular injection

## Abstract

Osteoarthritis (OA) is a prevalent debilitating age-related joint degenerative disease. It is a leading cause of pain and functional disability in older adults. Unfortunately, there is no cure for OA once the damage is established. Therefore, it promotes an urgent need for early detection and intervention of OA. Theranostics, combining therapy and diagnosis, emerges as a promising approach for OA management. However, OA theranostics is still in its infancy. Three fundamental needs have to be firstly fulfilled: *i*) a reliable OA model for disease pathogenesis investigation and drug screening, *ii*) an effective and precise diagnostic platform, and *iii*) an advanced fabrication approach for drug delivery and therapy. Meanwhile, microfluidics emerges as a versatile technology to address each of the needs and eventually boost the development of OA theranostics. Therefore, this review focuses on the applications of microfluidics, from benchtop to bedside, for OA modelling and drug screening, early diagnosis, and clinical therapy. We first introduce the basic pathophysiology of OA and point out the major unfilled research gaps in current OA management including lack of disease modelling and drug screening platforms, early diagnostic modalities and disease-modifying drugs and delivery approaches. Accordingly, we then summarize the state-of-the-art microfluidics technology for OA management from *in vitro* modelling and diagnosis to therapy. Given the existing promising results, we further discuss the future development of microfluidic platforms towards clinical translation at the crossroad of engineering and biomedicine.

## Introduction

Osteoarthritis (OA) is one of the top rising disability-causing conditions worldwide. In 2020, the global prevalence of knee OA was 22.9% in individuals aged over 40 and it increases in correlation with the individual's age 1, 2. As a whole-joint disease, OA affects synovium and subchondral bone in addition to articular cartilage. Responding to the altered joint microenvironment, the bone-cartilage architecture remodels *via* cell adaptations, including abnormal chondrocyte differentiation 3 and senescence 4. Being a multifactorial disease with complex pathophysiology, OA exhibits heterogeneous clinical presentations, posing challenges to the current clinical diagnosis and management strategies.

Generally, the existing OA management strategies can be categorized into nonpharmacologic and pharmacologic strategies. Nonpharmacologic improvements include lifestyle modifications such as physical exercise 5 and weight loss 6. As for pharmacologic interventions, examples are acetaminophen and nonsteroidal anti-inflammatory drugs (NSAIDs) 7, health supplements, such as glucosamine and chondroitin 8, or by surgical means, the total joint replacement 9. From mild to severe symptoms, the clinicians will decide to use a combination of these methods to treat patients according to the actual situation. The total joint replacement surgery is the very last approach and will only be performed on patients whose symptoms have not improved by other treatments. So far, all of these strategies focus on relieving pains and improving the life qualities after the onset of OA. There is no intervention nor cure to restore the structural defects in the joint.

As a prevalent degeneration disease with an increasing population of patients, critical improvement in current OA management strategies is highly demanded. A combinational approach termed theranostics is one of the promising candidates for the next-generation OA management strategies. By definition, theranostics is a strategy that combines therapeutics with diagnostics 10. It consists of a diagnostic test that identifies the disease state of the patient followed by targeted drug therapy based on the diagnostic test result. To date, it has been successfully applied to cancer therapy 11. Compared to traditional approaches, theranostics demonstrates a higher therapeutic efficacy and reduced adverse effects in curing cancer. Inspired by its success in cancer treatment, a similar methodology can be adapted to OA management strategies. However, theranostics for OA is still at the early stage of development with only a limited number of studies reported 12-16. To boost the development of OA theranostics, several fundamental questions shall be firstly addressed: *i*) a reliable OA model for disease pathogenesis investigation and drug screening, *ii*) an effective and precise diagnostic platform, and *iii*) an advanced fabrication approach for drug delivery and stem cell therapy.

The microfluidic technology, the manipulation of unique fluidic properties at the micrometre scale 17, 18, provides potential solutions to the obstacles for OA theranostics. As a versatile and powerful platform, the microfluidic technology has been applied to a wide range of different fields including disease modelling and drug screening 19, biosensing 20, and biofabrication 21, 22. However, little attention has been paid towards employing the technology for OA. Hence, this review aims to bridge this gap by introducing the pathogenesis and management of OA and how microfluidics might contribute to the aforementioned three aspects: OA modelling and drug screening, diagnosis, and therapy. Although limited achievement is reported for microfluidics-based OA theranostics, the individual success of microfluidics-based systems in these three aspects could pitch in the future development of OA theranostics. In this review, we first introduce the basic pathophysiology of OA. Then, the state-of-art developments in microfluidics for OA *in vitro* modelling, diagnosis, and therapy are discussed. Through the analyses of the existing stage of the microfluidic technology on OA management, an in-depth outlook for its future development is given at the end of this review.

## The Painful Joint: Pathophysiology of OA and Current Challenges

Instead of simple wear and tear disorder in the cartilage, evidence suggests that OA should be conceived as an inflammatory disease 23. Collectively, proinflammatory cytokines secreted by hypertrophic and apoptotic cartilage chondrocytes activate bone-remodelling osteoclasts and synovium macrophages, leading to systemic inflammation in OA. As a result, protease expression is upregulated, causing the degradation of collagen responsible for maintaining the joint structure.

In healthy joints, the articular cartilage covers the end of the bone forming the joint. This smooth structure acts as a protective cushion, allowing bones to glide with minimal friction and reducing mechanical damage resulting from severe loading 24. OA occurs when this protective cartilage cushion gradually deteriorates. As cartilage starts to erode, pathological changes occur in the tissue lining known as tidemark, which separates the zone of calcified cartilage from the non-calcified upper cartilage areas 25. The tidemark repeatedly doubles and develops spikes of fibrous tissue, and most of the time, these spikes grow into the non-calcified articular cartilage 26.

When the cartilage degradation progresses, the fibrous tissue undergoes endochondral ossification, and the cartilage is replaced with bony tissue outgrowths. The bony outgrowths at the margins of the joint are also referred to as osteophytes 27. Previous clinical studies have reported that cartilage destruction positively correlates with the degree of osteophyte formation 28 and that formation of osteophyte is more closely associated with pain than the rate of joint space narrowing 29. This process is also coupled with the growth of new blood vessels that could penetrate the thinning cartilage to reach the articular surface. Chondrocytes produce mediators such as cytokines and chemokines, associated with synovial inflammation even during the early stages of osteoarthritis 30. The number of synovial mast cells increases and the presence of auto-antibodies has been reported 31. Synovial inflammation around cartilage lesions can further accelerate cartilage damage resulting in a vicious cycle.

In severe stages of osteoarthritis, osteonecrosis leads to radiographically visible morphological changes in the subchondral bone. There is a significant increase in bone turnover and remodelling during the disease progression, which primarily results from osteocyte dysfunction 32. Bone homeostasis regulating bone-forming osteoblasts and bone-resorbing osteoclasts is disrupted. The cortical subchondral plate thickens, and the trabecular bone becomes increasingly irregular.

Due to the complexity of OA pathophysiology, it poses great challenges to the current diagnosis and management strategies. Specifically, these challenges are the lack of disease modelling and drug screening platforms, early diagnostic modalities, disease-modifying drugs, and effective delivery approaches.

### Insufficient disease modelling and drug screening platforms

As a complex degenerative disease, the cellular and molecular mechanisms of OA onset and progression are still not clearly understood. In addition, due to the heterogeneous nature of the disease, currently, no single model can reflect its overall pathogenesis. Thus, different models are selected based on the intended studied aetiology and purpose, which can be broadly categorized as two-dimensional (2D) and three-dimensional (3D) models. 2D culturing models grow cells on flat surfaces, which are quick, easy to work with, but exhibit reduced capability to investigate cell-cell and cell-extracellular matrix (ECM) interactions. Static 3D models grow cells in biomimetic tissue scaffolds and better recapitulate the microenvironment of human tissues. However, they still lack biological complexity, and generate results that do not often complement animal models 33. To date, the use of animal models is essential for preclinical discoveries, from fundamental to translational research. Considering the ethical issues, extensive time span of drug discovery and genetic difference between human and animals, there is a pressing need for the development of novel, physiological, dynamic 3D cell culture models to provide alternatives to *in vivo* studies.

### Lack of early diagnostic modalities

Clinically, the diagnosis of osteoarthritis is usually determined by radiographic evaluations. Changes such as osteophyte formation, narrowing of joint space, presence of subchondral cysts and sclerosis, and joint deformity can be indicators of osteoarthritis 9. These changes can result in joint symptoms such as pain, stiffening, and loss of function. They also vary with time, joint sites and individuals. Incidence and prevalence are therefore difficult to determine with current diagnosing modalities. Currently, a clinical diagnosis can only be made when the patient presents symptoms 34. There is a problem of using symptoms to define the progression of OA since such symptoms only develop towards the late stage of the disease and are probably irreversible. Moreover, symptoms fluctuate substantially over time and are influenced by concurrent pathology and pain pathway modulation 35. Therefore, symptom-based diagnosis has limited value in the early prevention of OA.

### Lack of disease-modifying drugs and effective delivery approaches

Until now, no drugs are available that have shown a significant disease-modifying effect in the clinical studies that improve both symptoms and structural defects in OA. Commercially available pharmaceutical agents target symptom control only. Chondroitin and glucosamine show anti-inflammatory and anticatabolic properties *in vitro* 8, yet their abilities to relieve symptoms or delay the structural progression of osteoarthritis in clinical trials have been conflicting. Similarly, hyaluronic acid (HA) has been widely used as viscosupplementation administered *via* intra-articular injections to compensate for the lowered lubricant in the synovium 36, but no clinically relevant benefit has been proven in terms of pain or function 37, and no convincing evidence of structural benefit is available. One of the important reasons for the inconsistent performance between *in vitro* studies and clinical trials is delivery and retention of the drugs inside the joint. Insufficient delivery of the target molecules to the joint and fast degradation of the active ingredients are believed to affect the clinical performance of those OA-modifying drugs 38.

## Investigating OA on the Bench: *in vitro* Microfluidic Models of OA

As a multifactorial disease, the exact pathogenesis of OA is rather complicated to study. Having a disease model on the bench is critically important to uncover the development of OA. Although *in vivo* models using animals are so far the most relevant preclinical models of OA, they are usually cost-intensive and time-consuming. Besides, animal testing has been long criticized for its ethical concerns. On the other hand, *in vitro* models based on *ex vivo* tissue samples or 2D cell cultures provide a simpler approach and have a lower time cost 39. However, existing *in vitro* models are oversimplified and fail to recapitulate the complicated pathophysiology of the whole joint. The influence of the microenvironment is largely lost in the 2D cell culture models and the role of external factors such as mechanical loading cannot be studied in static 3D *in vitro* models. Therefore, an advanced *in vitro* OA model with high fidelity and precise control over the microenvironment is highly demanded.

Organ-on-a-chip (OOC) devices have emerged as the next-generation *in vitro* models that fill the gap between the clinical trials and the conventionally used preclinical *in vitro* models. An OOC device is a microfluidics-based 3D cell culture platform that mimics the essential functional aspects of specific organs or tissues 40-44. Compared to traditional 2D *in vitro* models, OOC devices, as a 3D cell culture platform, better preserve the organ structural fidelity and incorporate the influence of the 3D microenvironment. Benefiting from microfluidics, OOC devices are able to provide a dynamic culture environment as well as other environmental controls such as mechanical stretching 45 and electrical stimulation 46. Moreover, they can be further integrated with biosensors to allow continual monitoring 47, 48. Therefore, they are promising *in vitro* tissue or organ models allowing us to study the development of diseases and screen the efficacy of drug candidates 49. To date, a variety of OOC devices have been developed to model different organs including the lung 45, 50, liver 51, 52, heart 41, 53, 54, brain 55, 56, skin 57-59, blood vessel 60, 61, and tumour 62, 63. There are quite a number of OOC devices that have been even successfully translated into commercial products 64, 65. Recently, OA, the whole-joint disease, attracts growing attention from the OOC field. Piluso *et al.* provided a comprehensive review on the state-of-art development of *in vitro* models for the articular joint, with an emphasis on 3D OOC devices 66. In this review, we specifically focus on the emerging interest from OOC field to modelling OA. Within the past 2 years, several OOC devices have been developed for creating *in vitro* OA models. These OA-on-a-chip devices focus mainly on two thrusts: disease modelling and drug screening.

### Disease Modelling

As a whole-joint disease, the development of OA is associated with an array of different factors including cartilage degeneration, abnormal subchondral bone remodelling, whole-joint inflammation, and joint overloading. One of the major advantages of the OA-on-a-chip devices as an *in vitro* model is that they allow us to study one specific factor at a time and minimize the interference that may be present in *in vivo* studies. Enabled by the microfluidic technology, several OA-on-a-chip devices have been developed to model the influence of osteochondral interface, mechanical loading, and inflammation on disease development.

**Figure 1:**
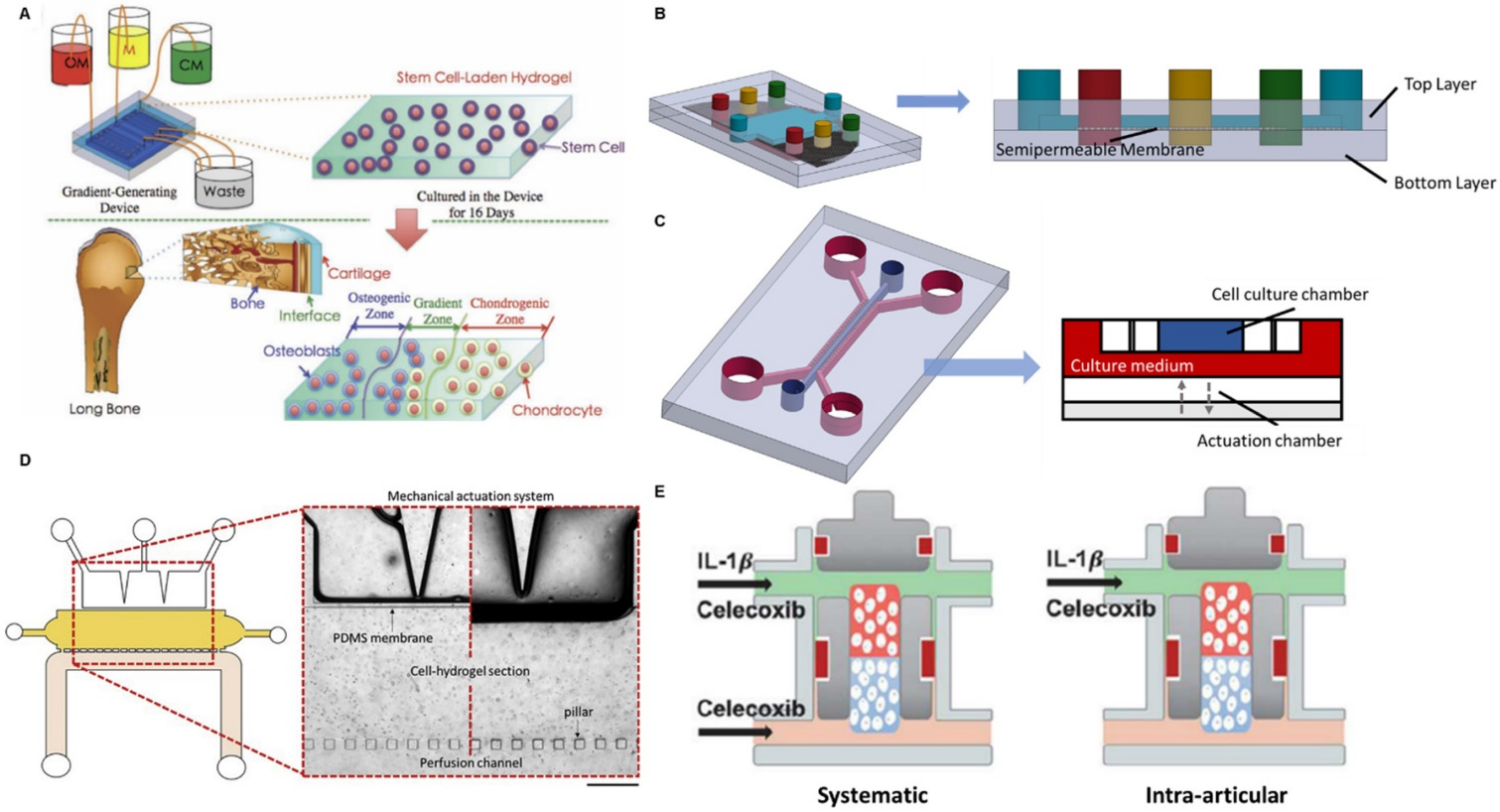
** Schematics of OOC-based platforms for OA modelling and drug screening. (A)** A device mimicking the osteochondral interface. Three different culture media were perfused into the device to create a gradient translational phase from an osteogenic zone to a chondrogenic zone. The stem cells encapsulated in the corresponding area were differentiated into osteoblasts and chondrocytes, representing the bone and cartilage. Adapted with permission from 76, copyright 2013 John Wiley and Sons. **(B)** A device studying the fluid shear force and bone-cartilage crosstalk. Chondroinduced and osteoinduced stem cells were seeded on top and bottom layer separately. The fluid shear force exerting to the cells was controlled by the fluid flow rate. **(C)** A device simulating the mechanical loading in the knee joint (left). A cross-section of this multi-chamber OOC device (right). The actuation chamber was regulated by an electro-pneumatic system to generate mechanical loading to the cell culture chamber. **(D)** A device studying the effect of mechanical loading and blood vessels on OA development. Zoomed in image: A mechanical actuation chamber simulating loading in the joint (top). A cell culture chamber of chondrocyte representing cartilage (middle). A perfusion channel supplying culture medium mimicking blood vessels (bottom). Adapted with permission from 71, copyright 2020 Elsevier. **(E)** A device studying systemic administration and intra-articular delivery of OA drugs. A cell-culture chamber was placed in the middle of the device with a cartilage region (red) and a bone region (blue). The upper supplying channel represented the synovial fluid and the lower supplying channel represented the blood vessel. Adapted with permission from 75, copyright 2019 Frontier Media.

The articular joint contains both the cartilage and the bone. The well-being of the osteochondral interface between these two tissues is disrupted in OA and consequently affects the signalling and nutrient exchange across the tissues 67. Therefore, an *in vitro* model recapitulating the interactions between cartilage and bone will enable an in-depth study on the role of the osteochondral interface in OA development. To achieve that, an OA-on-a-chip platform mimicking osteochondral interface was developed by a gradient-generating microfluidic system 68. It was a two-layered compartmentalized device: a bottom layer to culture mesenchymal stem cells (MSCs) in agarose hydrogel and an upper layer containing three independent microchannels (**Figure 1A**). A porous membrane was sandwiched in between the two layers so that the fluids in the upper channels could diffuse to the bottom layer. Through pumping in three different culture media (chondrogenic differentiation medium (CM), normal medium, and osteogenic differentiation medium (OM)) into the three microchannels, a gradient of culture environment from chondrogenesis to osteogenesis was created as a result of media diffusion. Consequently, MSCs in the bottom layer showed a distinct gradient differentiation distribution: in the distal CM-influenced side, the cells expressed chondrogenic markers; in the distal OM-influenced side, the cell-expressed osteogenic markers. A cartilage-bone interface was hence generated. Such a simple gradient-generating microfluidic device demonstrates the potential applications in modelling interfacial tissues. The incorporation with microfluidics further enables precise control on both the pattern and the composition of the gradient. A more delicate two-layered microfluidic device for modelling the osteochondral interface was developed in a more recent study to investigate the effect of shear force and bone-cartilage crosstalk in OA 69. A larger rectangle upper chamber was created to culture osteo-induced MSCs while the bottom layer was combined with three independent channels to culture chondrocytes, chondral-induced MSCs and a mixture of these two types of cells, respectively (**Figure 1B**). The crosstalk between bone and cartilage was hence established by such a spatial arrangement. Since the microfluidic device provided a dynamic culture environment with the interstitial flow in the superficial zones of articular cartilage, the authors were able to study the bone-cartilage crosstalk under shear stress created by the fluid-induced mechanical stimulus. It was reported that the dedifferentiation marker collagen I expression in the cartilage layer increased under shear stress, while the introduction of osteo-induced MSCs in the upper layer could offset this change 69. These results indicate that osteo-induced MSCs have a significant rescue effect on dedifferentiation and are able to maintain the integrity of the joint.

Having load-bearing as the major function, mechanical loading plays a critical role in OA development. A cartilage-on-a-chip model was fabricated to explore the impact of joint overloading on OA 70. Another two-layered OOC device was developed in the study: an upper layer for the 3D culture of healthy primary human chondrocytes and a designated bottom layer as the actuation compartment generating cyclic loading *via* confined compression (**Figure 1C**). Simply through repeated compressions, the researchers successfully induced an *in vitro* OA phenotype characterized by increased expressions of cartilage catabolic genes and decreased expressions of anabolic genes. To better mimic the pathophysiological environment of the OA joint, the team added interleukin-1*β* to the culture medium during the rest phase of compression to induce inflammation. It was found that the inflammation induction caused the upregulation of OA-associated gene expressions.

Aside from mechanical loading, another OA-on-a-chip device was developed that simulated both the mechanical loading in the joint and nutrient transport from blood vessels 71. This microfluidic device contained three interconnected chambers representing mechanical loading, cartilage tissue and blood vessel respectively (**Figure 1D**). A cell-laden hydrogel culture chamber was located in the centre serving as the cartilage tissue model. On one side, the hydrogel was connected to a mechanical actuation section through a polydimethylsiloxane (PDMS) membrane, which induced compressive strains and shear stress. On the other side, it was connected to the perfusion channel by discontinuous pillars, which allowed nutrient transport across the channels. Despite the unique design, it was a rather preliminary study where only cell viability was tested. Given the three independently controlled channels, the investigation of OA phenotype under imbalanced loading (from different directions) and the effect of blood vessels could be achieved in this device in future studies.

### Drug Screening

Drug screening is another important application area of OA-on-a-chip devices. Compared to traditional drug screening platforms, OOC-based screening platforms have their own advantages in terms of easy manipulation, high fidelity in humanly responses, and the potential for scaling up and higher-throughput operations 43, 72, 73. Given these advantages, OOC-based screening platforms offer the possibility to replace the cost-intensive and time-consuming *in vivo* animal testing for the preclinical trials. Consequently, OOC-based drug screening platforms are likely to significantly speed up the drug discovery process by shortening the time cost in the preclinical trials. Given the fact that no disease-modifying drugs are available for OA, the application of OA-on-a-chip devices for drug screening falls into the interest of both pharmaceutical companies and orthopaedic professions.

As aforementioned, Occhetta *et al.* developed a cartilage-on-a-chip model mimicking the OA phenotype 74. Upon the completion of the model development, the authors examined the effects of several OA drugs currently in preclinical or clinical trials with known effects including dexamethasone, rapamycin, and celecoxib. The on-chip testing of dexamethasone demonstrated a similar response to clinical results with the reduced expression of matrix metallopeptidase-13 (MMP-13) and but no change in that of interleukin-8 (IL-8) after treatment. A dose-dependent effect of rapamycin was also observed on-chip where MMP13 was reduced at both doses but IL8 inhibition was only seen at the high dose. The non-steroidal anti-inflammatory drug, celecoxib, managed to suppress the expression of both MMP-13 and IL-8 on-chip as expected. After validating the platform *via* screening drugs with known effects, the authors also conducted a drug discovery experiment by testing the effect of a drug under development, HA alkylamine HYADD4. Compared to HA, HYADD4 demonstrated a stronger efficacy in reducing the expression of MMP-13.

To incorporate the influence of bone in the OA drug response, Lin *et al.* developed an osteochondral tissue chip to test OA drugs 75. It was a three-layered device where a cell culture chamber was sandwiched by two medium supply channels from top and bottom (**Figure 1E**). Induced pluripotent stem cells (iPSCs)-derived mesenchymal progenitor cells were encapsulated in the culture chamber with the upper half being differentiated into the cartilage tissue and the lower half being differentiated into the bone tissue. Celecoxib was selected as the benchmark drug to validate the platform. By pumping in celecoxib from both top and bottom supply channels, the authors were able to mimic the systematic administration of the drug. Intra-articular injection of the drug was also simulated by pumping the celecoxib from the top supply channel only. Noteworthily, the on-chip screening of celecoxib showed that systematic administration had a better therapeutic effect in disease-modifying than intra-articular injection.

Collectively, OOC-based drug screening platforms are a versatile tool that allows screening the efficacy of OA drugs at different doses and delivery routines. With the employment of iPSCs, this platform is ready to be fabricated in a patient-specific manner. Although most of the existing literature on OA-on-a-chip devices for drug screening emphasizes the validation of drugs with known effects, the promising results from this proof-of-concept stage demonstrate their potentials for drug discovery in the future.

## Diagnosing OA-on-a-Chip: Microfluidics-based Biosensor

Biomarkers are endogenous molecules that indicate or reflect a specific biological or pathological process, to identify the outcome or endpoint of the pharmacological response to a therapeutic intervention. These biomarkers could measure how the patient feels and functions. In OA, biomarkers reflect the disease process leading to whole-joint destruction, aiding in the understanding of the pathophysiology of disease and prediction of structural changes. **Table 1** summarizes a list of potential biomarker candidates for identifying patients at risk and allowing early diagnosis for better therapeutic efficacy. These biomarkers can be detected in the biological fluids, including serum, synovial fluid, urine, or the ECM of cartilage 77. During treatment, biomarkers for OA can help the stratification of patients with uniform biomarker characteristics, which could enable targeted therapies to specific patient groups. In drug development, OA biomarkers could help confirm drug efficacy as outcome parameters in preclinical and clinical studies and could be used to identify therapeutic doses and to evaluate the toxic effects of drugs in development to treat OA. As a whole-joint disease, the proper biomarkers for cartilage, bone, and synovium are equally important. A thorough understanding of the tissue-specific biomarkers will significantly improve the precision and specificity of OA diagnosis.

### Tissue-specific OA Biomarkers

#### Cartilage

In healthy joints, the articular cartilage is an avascular tissue with a slow remodelling process. The remodelling rate increases as OA initiates 102. Within the cartilage, chondrocytes are the dominant cell type. Cartilage structure and biochemical components are strictly regulated by chondrocytes in response to mechanical and chemical changes in the microenvironment 103. During disease progression, chondrocytes become hypertrophic 104. As a result, there is increased protease expression during the disease progression including matrix metalloproteinases (MMPs) and a disintegrin and metalloprotease with thrombospondin type I motifs (ADAMTS), cleaving structural components and increasing cartilage turnover 105.

Type II collagen is the main structural protein in the cartilage, serving as a network that receives stabilization from other collagen types and non-collagenous proteins, providing tensile strength within the cartilage 106. This framework keeps aggrecan and proteoglycans embedded in place. The early stage of the cartilage degeneration process starts from aggrecan degradation, followed by the catabolism of collagen fibres, resulting in loss of cartilage integrity 107. Several cleavage products from the proteolytic burden associated with OA have been identified: MMP-derived C-telopeptide of type II collagen fragment CTX-II 108, nitrated type II collagen degradation fragment (Coll2-1 NO2) 109, and aggrecan ARGS neo-epitope fragment from aggrecan degradation 110. Type II collagen can be cleaved by collagenase-generated cleavage neoepitopes, for example, C2C, C1 and 2C 111.

Aggrecan also contains many chondroitin sulphate and keratan sulphate (KS) chains 112, they are responsible for binding water molecules to create a swelling pressure causing a compressive stiffness that resists deformation and compression of cartilage in the network. Early OA articular cartilage destruction starts with glycosaminoglycan (GAG) release from articular cartilage surfaces 113. Chondroitin sulphate 846 epitopes (CS846) is a by-product of proteoglycan metabolism, which is increased during OA 114.

Cartilage oligomeric matrix protein (COMP) is an ECM glycoprotein member of the thrombospondin family of calcium-binding proteins. It is believed to have a structural role in endochondral ossification and the assembly and stabilization of the ECM by its interaction with collagen fibrils and matrix components 115. COMP is also found associated with fibril formation of collagen types I and II by promoting the early association of collagen molecules 116. Combination detection of CS846 and COMP can effectively reflect the degradation of cartilage and synovial tissues 89.

YKL-40, also known as chitinase-3-like protein 1 or human cartilage glycoprotein 39, can be secreted by several different cell types in the joint tissue, including macrophages, articular chondrocytes and synoviocytes 117. Its expression is upregulated during chondrocytes differentiation and is associated with synovial inflammation 118.

Cartilage homeostasis relies on the regulated catabolism of matrix proteins and proteins synthesized by chondrocytes. The major protein responsible for cartilage synthesis is produced as a procollagen with a propeptide domain 119. N-terminal propeptide of collagen IIA (PIIANP) is one example of such a product. It is synthesized during OA as a repair attempt to compensate the cartilage degradation 120. A low level of PIIANP is associated with OA progression 92. Similarly, decreasing level of procollagen type II C-propeptide (PIICP) indicates joint space narrowing and reduction of articular cartilage thickness in OA 94.

#### Bone

As a dynamic tissue, bone is constantly remodelled in response to mechanical loading, metabolic changes and microdamages 121. In a healthy joint, bone remodelling is well-balanced between bone resorption performed by the osteoclasts removing old or damaged bone and bone-forming osteoblasts replacing the new bone 122.

In the bone matrix tissue turnover, collagen type I is degraded by the cysteine protease cathepsin K secreted by osteoclasts during bone resorption. As a result of degradation, C-terminal telopeptide of collagen I (CTX-I) and N-terminal telopeptide of collagen I (NTX-I) are produced. Both expressions are increased in progressive OA when bone resorption increases to predict joint space narrowing 95. CTX-I is also found associated with the presence of osteocytes 123. Pyridinoline (PYR) and deoxypyridinoline (D-PYR) are non-reducible crosslinkers of mature collagen. PYR is found in most collagenous tissues including cartilage and bone, while D-PYR is more restrictive and only found in the bone 124. The presence of PYR and D-PYR indicates bone metabolism 79.

For bone formation, two proteins are potential biomarkers. Osteocalcin (OC) is a major non-collagenous protein in bone, containing three *γ*-carboxyglutamic (Gla) acid residues. It is a marker of mature osteoblasts as well as bone formation. While its role in the pathology of OA is not fully known, it has been reported to predict OA progression 97. Bone sialoprotein (BSP) is a major non-collagenous ECM protein that belongs to the small integrin-binding ligand N-linked glycoproteins (SIBLING) gene family 125. BSP can be expressed by mature osteoblasts, osteoclasts and hypertrophic chondrocytes of the growth plate under pathological conditions. It could be a key mediator of the hypertrophic chondrocytes-induced angiogenesis 98.

#### Synovium

The synovial membrane is a thin cellular structure, which encapsulates the joint cavity from the external endothelial cell structures. During inflammation, the synovium is filled by immune cells, which induce fibrosis and neovascularization, also known as synovitis. This causes macrophages to produce pro-inflammatory cytokines resulting in proteolytic burden 126.

Glucosyl-galactosyl pyridinoline (Glc-gal-PYR) is a glycosylated analogue of pyridinoline, found abundant in synovium tissues. Its expression has shown a strong correlation with symptoms of OA. Glc-gal-PYR is released during synovium degradation, and is absent from bone and cartilage, making it a distinctive potential biomarker of OA 99. HA is a non-sulphated glycosaminoglycan found in synovium maintaining the viscoelasticity of joints. HA can reduce the production of proinflammatory mediators and nerve impulses and nerve sensitivity associated with OA pain 127. Inflammatory synovial fluid contains a large amount of type III procollagen (PIIINP), which are released into the blood during fibrogenesis. It is a direct indicator of collagen synthesis and synovium synthesis 101.

### Microfluidics-based Biosensors for OA Diagnosis

**Figure 2:**
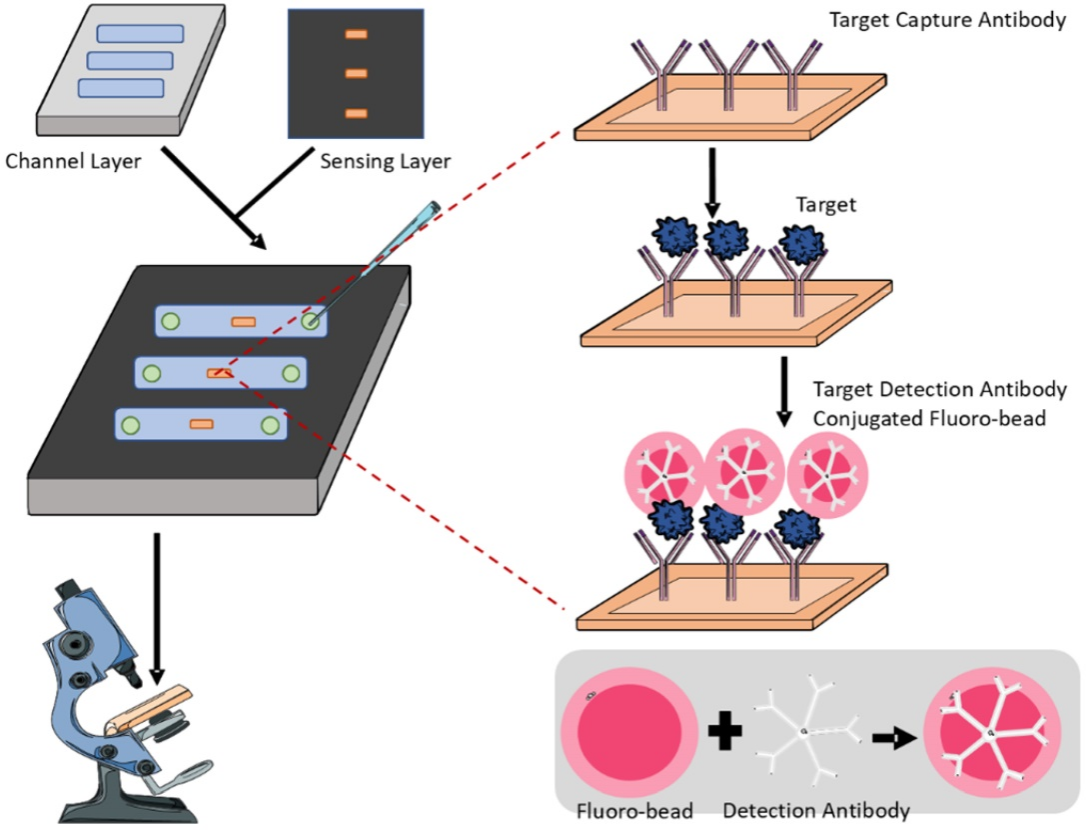
** Representative schematics of an antigen-antibody microfluidic biosensor.** The biosensor consists of a bottom channel layer and a top sensing layer. The former allows loading of bio-fluids while the latter detects the presence of targeted biomarkers. The sensing layer contains capture antibodies specific to the target biomarker and fluorescent antibodies for visualisation and quantification.

Biomarker-based detection is generally simpler and faster than tissue examination or radiographic diagnostic methods. To overcome the limitations of conventional diagnostic modalities, the combination of sensitive biomarkers with microfluidics enables the generation of high-throughput screening with reduced labour cost, allowing OA to be diagnosed at early stages for better therapeutic interventions. Generally, microfluidic biosensors for OA diagnosis adopt an antigen-antibody binding mechanism. The surface is coated with an antibody against the targeted biomarker, and depending on the obtained positive signals, the level of biomarker from the sample fluid can be determined (**Figure 2**). Due to the inherent properties of microfluidics, only a small volume of samples is required without sacrificing sensitivity and specificity 20. Besides, such a design of a microfluidic sensor is so versatile that it can be easily modified for the detection of different biomarkers by manipulating the antibody coating during fabrication.

According to the biomarker candidates listed in **Table 1**, several microfluidic devices have been developed to detect either cartilage-specific or synovium-specific biomarkers from different biological fluids including serum, synovial fluid, and even urine. A fluorescent microbead guiding chip-based immunoassay to detect COMP in serum and synovial fluidic is developed by Yoon's team in 2012 128. The COMP-detecting antibody-conjugated fluorescent microbeads were applied on four immunoreactive regions in the device, each with five patterns, to allow multiple assays. The binding signals could be directly analysed under a microscope. In 2015, the same team developed another microfluidic device to detect CTX-II 129. Samples from both serum and urine containing CTX-II could be treated with CTX-II antibody-conjugated fluorescent microbeads and simultaneously analysed on the same chip. Then, the immune-specific signal could be quantitatively calculated by counting the number of fluorescent microbeads from the observed images to determine if cartilage degradation occurred and to diagnose OA. The device was improved from the previous example, allowing samples from different body fluid to be assessed simultaneously.

Schulte and Suginta fabricated the microfluidics-based immunosensor to detect human cartilage chitinase-3-like protein 2 (hYKL-39) expression in the synovial fluid 130. hYKL-39 is a human cartilage chitinase-3-like protein 2 131. Anti-hYKL-39 was fixed to a thin but insulating self-assembled monolayer of thiourea/1-dodecanethiol on gold electrodes in the device. The hYKL-39 expression could be quantified by calculating the exponentially decaying capacitive current responses of hYKL-39 immunosensors to potential sweeps. Capacitive hYKL-39 immunosensing has shown a lower detection limit than the enzyme-linked immunosorbent assay (ELISA) used for result validation.

Apart from designing and fabrication of the microfluidics-based biosensor, validation of the device is equally important to ensure the diagnostic accuracy, also known as quality control. The common validation method involves correlating the obtained result from microfluidic with that in the conventional immunoassays, for example ELISA 132, to determine the amount of proteins present. Due to high sensitivity and specificity, ELISA has been widely used for quality control against other newly developed assays 133, including microfluidics-based assays.

The materials used for fabricating microfluidics-based biosensor is another vital consideration. PDMS is the most popular material of choice with attractive properties, including low cost, ease of use, and optical clarity 134. Moreover, it allows for rapid prototyping of microscale devices 135. These properties enabled many laboratories to quickly develop novel devices for their research. However, in microfluidics-based biosensors, adsorption and absorption of biomarkers can occur. PDMS is a permeable material susceptible to adsorption and absorption of bulk hydrophobic substances 136. As such, the loss of biomarkers will affect the outcome in a biosensor during diagnosis. This can be overcome by coating the PDMS surface with impermeable material during the fabrication. Studies showed that PDMS coated with parylene 137 and paraffin wax 138 can effectively prevent the absorption of small molecules. Alternatively, other materials, such as thermoplastics, ceramics, and resin, can be used for fabricating biosensors as proposed 139-141.

## Curing OA at the Bedside: Microfluidics for OA Therapy

Despite having been long-studied, there are limited clinically approved strategies for OA therapy at the bedside. Instead of rescuing the joint degeneration, existing clinically approved treatment focuses primarily on pain medication and anti-inflammation 142. Given the nature of the traditional OA therapies, the therapeutic efficacy for late-stage OA is far from satisfactory. In recent years, different new therapeutic strategies for OA have been developed, with intra-articular injection and cytothereapy being the most promising novel OA therapies 143. Meanwhile, microfluidics, as an emerging biofabrication strategy, demonstrates its potential in significantly improving the clinical performance of these two therapies.

### Intra-articular Drug Delivery

Intra-articular drug delivery, by definition, is the direct injection of drugs into the knee joint. Compared to oral administration of drugs, this localized treatment minimizes the systematic off-target effects and adverse effects such as gastrointestinal complications 144. Since drugs are directly injected into the inner space of the knee joint cavity, they have a higher local bioavailability to the articular cartilage, which, in return, reduces the doses required compared to the systematic delivery. However, the clinical performance of intra-articular injection is still far from satisfactory. Repeated intra-articular injection is usually required to sustain the efficacy. The main reason for this limitation is the short drug residence time in the joint cavity. Typically, the injected drug will be rapidly cleared from the synovial fluid by lymphatic drainage at the scale of hours, depending on the size of the molecules 145. Therefore, extending the drug retention inside the cavity after injection is critically important to improving the clinical performance of intra-articular injection.

Instead of injecting the drug molecules directly, encapsulating drugs into micro/nano carriers is an effective strategy to enable controlled drug release and extend drug retention 146. Typically, the drug molecules are encapsulated in a functional hydrogel scaffold, which can be designed for passive or active drug release 147. Rather than bulk hydrogels, drug molecules are more commonly encapsulated in hydrogel droplets, also known as microgels. This is because that *i*) the small dimension of microgels provides a better injectability even with small needles and *ii*) microgels are inherently modular and can be mixed for a diverse composition 148. Conventionally, microgels are fabricated by extrusion fragmentation 149, batch emulsion 150, electrospraying 151, and lithography 152. However, these methods have at least one of the following limitations: *i*) large variation in the size of microgels, *ii*) lack of control on the shape of the microgels, *iii*) low throughput in fabrication, and/or *iv*) sophisticated experiment setup. As an emerging and versatile biofabrication platform, microfluidics manages to overcome the aforementioned limitations and fabricates the drug carriers with designated structures in a high-throughput manner. Generally, the microgel formation mechanism in microfluidics can be categorized into two classes, passive and active methods 153. While the active methods are more widely used for liquid manipulation, the passive droplet formation approach is the major microfluidics for generating microgels. In the passive method, the droplet is formed by the emulsion of two immiscible dispersed and continuous fluids at the microfluidic junction. There are three common designs of the junction, including co-flow, cross-flow, and flow-focusing junction. Compared to conventional strategies, microfluidic emulsion generates a wide range of microgel sizes with low variation. By simply adjusting the flow rate of the oil or aqueous phase, the size of the microgels can be fine-tuned. Besides, the productivity can be easily levelled up by stacking multiple chips or integrating multiple channels 154. Furthermore, microfluidic emulsion has been successfully used to generate complex microgel structures such as Janus microparticles 155 and core-shell microgels 156. Given the advantages of microfluidics in fabricating microgels, it is a promising platform to improve the clinical performance of intra-articular injection from the aspect of material preparation.

**Figure 3:**
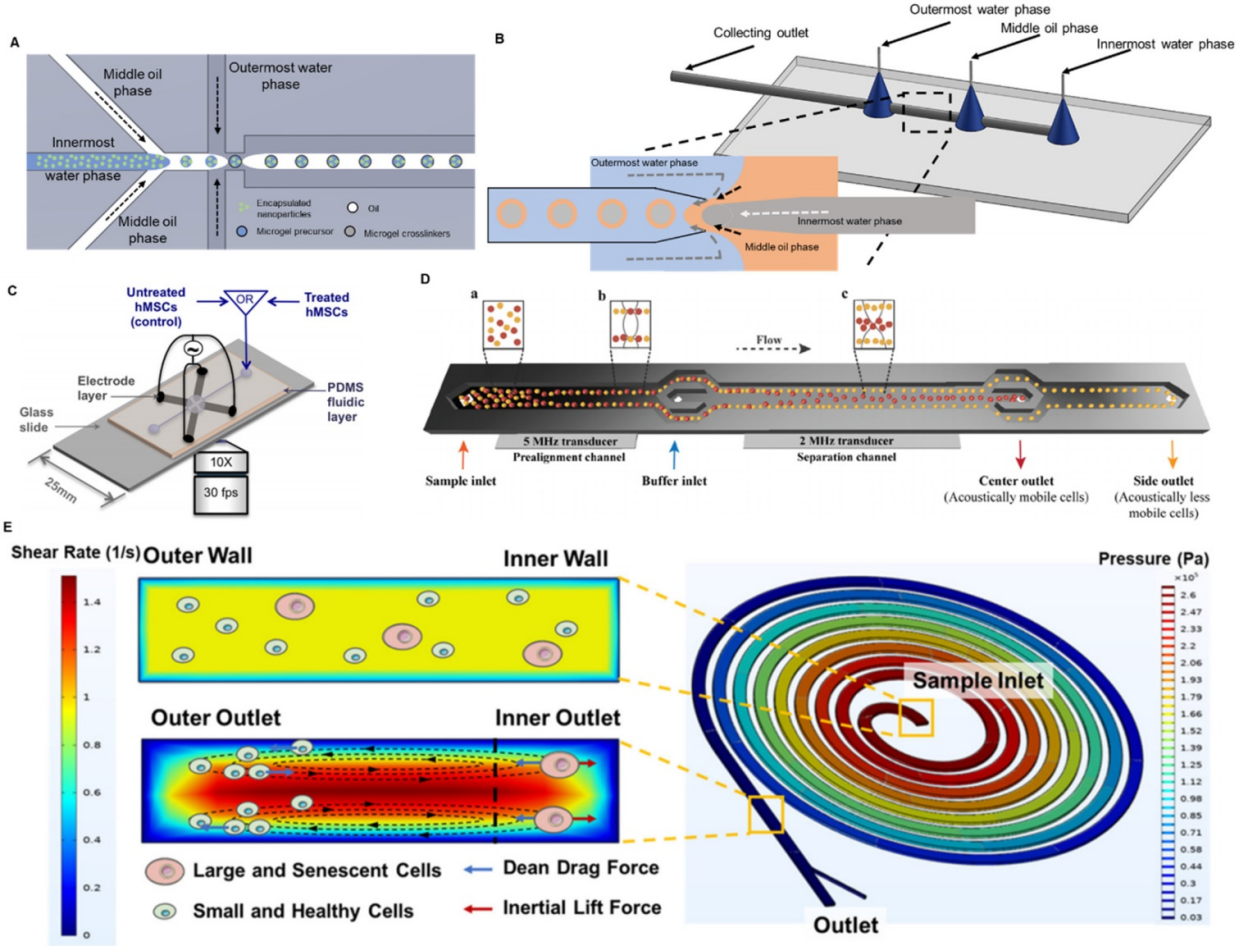
**Microfluidics for OA therapy. (A)** Flow focusing droplet microfluidics for fabricating nanocomposite microgels. The nanoparticles were suspended in the innermost water phase and hence encapsulated in the microgels. **(B)** Glass capillary microfluidics for fabricating core-shell microgels. Zoomed in figure: the core-shell microgels was generated at the junction of the three phases through double emulsion. **(C)** A microfluidics-based DEP platform for isolating MSCs. The MSCs were attracted to the four electrodes when the electrical field was on. Adapted with permission from 174, copyright 2014 AIP Publishing. **(D)** A microfluidics-based acoustophoresis platform for isolating MSCs. An acoustic wave with higher frequency was applied near the inlet to align the cells. An acoustic wave with lower frequency was applied in the center of the device to separate MSCs with different acoustical mobility. Adapted with permission from 175, copyright 2021 John Wiley and Sons. **(E)** Spiral microfluidics for eliminating senescent MSCs. Balanced by Dean drag force and inertial lift force, large and senescent MSCs were focused to the inner outlet of the device and the small and healthy MSCs were focused to the outer outlet of the device. Adapted with permission from 178, copyright 2021 AIP Publishing.

So far, microfluidics has been successfully utilized to fabricate drug carriers and lubricants for intra-articular injection. To achieve the sustained delivery of small molecules inside the knee joint, Castro *et al*. developed a nano-composite microgel system *via* microfluidic emulsion 157. Using a modified flow focusing droplet microfluidic device (**Figure 3A**), the authors encapsulated poly(lactic-co-glycolic) acid (PLGA) nanoparticles inside 50-μm poly(ethylene glycol) (PEG) microgels with a coefficient of variation lower than 6.5%. By conjugating tissue-targeting peptides to PEG, the microgels were able to specifically bind to the articular cartilage. Moreover, the *in vivo* study on rats showed that these microfluidics-fabricated microgel-based carriers were retained in the knee OA joint for more than 3 weeks. Moreover, hydrophobic drugs can also be easily encapsulated in microgels and sustainably released to the knee joint. To do that, Yang *et al.* first loaded the hydrophobic chonroinductive molecule, kartogenin, to liposome and then encapsulated the drug-loaded liposomes inside gelatin methacryloyl (GelMA) microgels through physical network hindrance and non-covalent interaction 158. A simple flow focusing microfluidic design was applied to generate these microgels with a diameter of 100 μm. After being injected into the mouse's knee joint, the residence time of the kartogenin-loaded liposome was extended from 15 days to 35 days. Such a liposome-anchored microgel system not only addresses the low solubility of kartogenin in an aqueous solution but also extends its retention time. Furthermore, microfluidics enables the co-encapsulation of both hydrophobic and hydrophilic drugs in a single microgel. Core-shell PLGA microgels were fabricated through water-in-oil-in-water emulsion in a glass-capillary microfluidic device as shown in **Figure 3B** 159. The hydrophobic kartogenin was encapsulated on the shell of the microgels whereas the hydrophilic chemokine, stromal cell-derived factor-1 (SDF-1), was encapsulated inside the core. Both molecules encapsulated were characterized to sustainably release for over 2 months. Apart from being a drug carrier, microgels themselves are inherently an effective lubricant 160. Due to the hydrophilic property of the hydrogel droplets, they could form a hydration layer for lubrication on the shell and consequently prevent the further deterioration of the knee OA. By dip-coating tissue-adhesive polymers onto the GelMA microgels, these microgels adhered to the surface of articular cartilage after injection and reduced the joint friction 161. Given the importance of local drug delivery and lubrication in treating OA, fabricating such a dual-function system will improve the efficacy of OA treatment. Recently, Zhang and his group developed a series of dual-function nanoparticles combining lubrication with reactive oxygen species (ROS) scavenging 162 or controlled local drug delivery 161, 163. Similarly, a dual-function microgel system was designed and fabricated with droplet microfluidics to enhance the performance of intra-articular injection 164. GelMA microgels were fabricated in a flow-focusing microfluidic device and coated with dopamine to strengthen the hydration layer. The anti-inflammatory drug, diclofenac sodium, was loaded to the lubricant microgels *via* physical absorption.

### Stem Cell Therapy

In OA management, intra-articular drug delivery is considered as an early preventative strategy to relieve the pain and, hopefully, delay the disease progression. Due to the absence of nerves and blood vessels, articular cartilage has a rather limited regenerative capability 165. At the late stage of OA, the damaged articular cartilage can barely be healed by drugs sorely. To halt the progression of OA *via* tissue regeneration, stem cells are a suitable candidate to effectively cure the disease even at the late stage. After being delivered to the knee joints, stem cells can not only differentiate into chondrocytes to regenerate the tissue but also secrete certain cytokines to suppress the inflammation 166. To date, there are two major MSC types used for OA cell therapy, bone marrow-derived stem cells (BMSCs) and adipose-derived stem cells (ASCs) 167. Although stem cells can be derived from other sources, BMSCs and ASCs are widely used for OA cell therapy due to their easy accessibility, free of ethical concerns, and high chondrogenic capability. A typical stem cell therapy involves, first, the isolation and purification of the cell source and then, the delivery of the cells. These two steps are equally important that would significantly determine the clinical performance of the therapy. Targeting the two steps, a variety of microfluidic devices have been developed to simplify the procedure of stem therapy, raise the throughput and consistency, and eventually improve the therapeutic efficacy.

As aforementioned, BMSCs and ASCs are the two main MSC types for OA stem cells therapy. Therefore, the first step in stem cell therapy is to isolate MSCs from bone marrow or fat tissues and purify them from other stromal cells in the tissues. Fernandes *et al*. comprehensively reviewed the existing approaches and mechanisms for stem cell isolation 168. Briefly, after harvesting cells from the corresponding tissues, slow-sedimenting MSCs are firstly separated from high-density cells and body fluids *via* centrifugation and then purified by plastic adherence, eliminating the non-adherent cells. The isolated MSCs are then expanded *in vitro* to prepare sufficient cell number for therapy. However, this simple isolation approach often ends up with an impure cell population after expansion 169, leading to unpredictable clinical performance. This is because that MSCs are inherently a heterogenous population with varying morphology and functionality 170. Senescent MSCs are a notable subtype characterized by their arrest of cell proliferation and reduced differentiation potential 171. The impurity of the cell source, in particular the presence of senescent MSCs, is believed to be a major factor affecting the therapeutic efficacy of stem cell therapy 172. Thus, additional isolation and purification strategies have to be implanted to achieve higher purity of active MSCs.

Fluorescence-activated cell sorting (FACS) is the gold standard in cell isolation with high resolution and throughput. FACS is a label-based method by which the target cells will be labelled by the fluorescent detecting antibody *via* a specific surface marker and be recognized and sorted by the machine. This process can be conducted in a simple Y-junction microfluidic chip 173. This FACS-on-a-chip platform provides a rapid response in sorting cells due to the laminar flow in the microchannel. The miniaturized device allows the high throughput sorting by simultaneously running multiple chips in parallel. Despite the high efficiency of FACS, its limitations as a label-based method are obvious. The use of detecting antibodies requires a throughout understanding of the surface marker of the target cells and increases the cost of operation. Moreover, the process of labelling could potentially bring undesired changes in cell functions 174. Hence, several different microfluidics-based label-free cell isolation approaches have been developed to isolate MSCs. Dielectrophoresis (DEP) employs the unique polarity and dielectric properties of each cell type and separates them with a controlled frequency of the alternating electric field. Adams *et al.* set up a DEP-on-a-chip platform by embedding four Ti-Au electrodes in a 2-mm-deep and 3-mm-wide PDMS-based microfluidic device as shown in **Figure 3C** 174. The group studied the DEP behaviours of human MSCs and observed heterogeneity within the cell populations. Such heterogeneity, in their opinion, originated from the difference in surface biomarker expressions, indicating the different cell fates of the MSCs. The dielectric signature of human MSCs characterized in this study laid the foundation for DEP-based label-free isolation of MSCs featuring different cell fates. Similarly, acoustophoresis utilized external ultrasound waves to distinguish cells with different acoustophysical properties. Generally, smaller cells with less density and stiffness tend to be acoustically less mobile and move slower in the channel. Olm *et al.* developed a 20-mm-long microchannel with two acoustic sources of different frequencies to purify the BMSCs (**Figure 3D**) 175. By acoustic isolation, the BMSCs collected from the side outlet were on average 3-µm smaller in diameter, 20% higher proliferation in rate and 6.65-fold and 2.9-fold higher in expressing stem cell pluripotency markers, *Nanog1* and *Oct4*, compared to the cells collected from the central outlet. More importantly, the microfluidic acoustophoresis did not compromise the differentiation capability of BMSCs into osteogenic or chondrocytic lineage.

Although DEP and acoustophoresis circumvent the process of labelling, they still require external power sources such as an electrical field or acoustic wave. Spiral microfluidics purely depends on the inertial focusing inside the microchannel, is a label-free size-based cell sorting without the need for external power sources. Balanced by Dean drag force and inertial lift force in the spiral microchannel, cells of different sizes will be focused to at different positions 176. Taking the advantages of size difference between BMSCs and other cells in the bone marrow, Lee *et al*. isolated mouse BMSCs from bone marrow samples using such spiral microfluidics 177. It is a rapid isolation strategy that 3×10^6^ bone marrow cells were sorted in 1 minute and retained over 95% viability. As aforementioned, the variation in the differentiation potential of the MSC source is the major cause for the inconsistent clinical performance of stem cell therapy. After isolating BMSCs from bone marrow samples, Chen *et al.* utilized a different spiral microfluidic device to further purify mouse MSCs 178. As the senescent MSCs with lower differentiation potential are larger in size, the authors effectively separated the senescent MSCs and normal MSCs into two outlets (**Figure 3E**). Taken together, spiral microfluidics can be used to effectively *i*) isolate the MSCs from other cells in the tissue and *ii*) eliminate the senescent MSCs with low differentiation potential. Compared to other microfluidic cell sorting methods, spiral microfluidics is simpler in experiment setup (no external power sources or labelling needed) and high throughput (isolating flow rate typical at several mL/min) with high post-isolation viability (>85%).

With the contributions from microfluidic technology, a high-quality stem cell source can be effectively prepared for cell therapy. Another challenge faced at the bedside for stem cell therapy is the delivery of cells. Direct injection of stem cells to the knee joint is apparently not an ideal approach 179. The high shear stress at the needle during the injection could be detrimental to the cells. Besides, low cell retention was also observed in delivering stem cells in saline 180. Therefore, a proper vehicle for stem cell delivery is highly desired. Similar to intra-articular drug delivery, droplet microfluidics effectively address the problem of cell delivery. In flow-focusing capillary microfluidics, BMSCs were encapsulated in GelMA microgels and injected into a rabbit to induce osteogenesis 3. The percentage of new bone volume was significantly raised by encapsulating BMSCs in GelMA microgels compared to direct injection with saline. Apart from encapsulating cells into the microgels, co-injection of MSCs and microparticle scaffolds was also proposed to enhance the *in vivo* delivery of stem cells 181. The author fabricated the hydrogel microparticles in flow-focusing microfluidics and then mixed these microparticles with MSCs right before the injection. Due to the annealing of microgels, a microporous hydrogel scaffold was formed *in situ* and the MSCs were trapped inside this scaffold. Co-injection of MSCs in microgels scaffold was found to promote cell migration and proliferation as well as extend the retention of MSCs in the subcutaneous implantation model. Although traditional stem cell therapy focuses on the delivery of individual MSCs, some recent studies showed that the delivery of multicellular MSC spheroids could further improve the therapeutic efficacy for OA 182, 183. In general, when cells aggregate into spheroids, their proliferation, matrix production, and differentiation capability are enhanced 184. As a result, MSC spheroids are expected to have a better regenerative capability than individual cells. Even though not yet being applied to growing MSC spheroids for OA therapy, microfluidics has been utilized to fabricate massive numbers of uniform spheroids in a time-efficient manner 59, 185, 186. A next-generation stem cell therapy could be thereby established by integrating multicellular spheroids and microfluidics.

## Outlook

With the global population ageing, OA has become an increasing social burden. Given the limited clinical performance of the traditional OA treatment, an improved theranostic strategy is strongly demanded. In this review, we analysed the contributions of microfluidic technology to this outstanding issue from benchtop modelling and screening to on-site diagnosis and bedside therapy. From all these aspects, the microfluidic technology has demonstrated its own advantages over the traditional approaches. Therefore, we believe that there will be further integration of microfluidics and OA theranostics in the next decade to come. In the future development of a next-generation microfluidics-based OA management, we envision that the following three trends will be the main focuses.

### The emerging role of organoids in disease modelling and therapy

Current approaches for *in vitro* modelling and cell-based therapy of OA still relies on the manipulation of individual cells. Different types of OA-related cells are sparsely encapsulated either in bulk hydrogels to build up an *in vitro* OA model or microgel vehicles for intra-articular injection. For *in vitro* modelling, this highly engineered tissue scaffold often loses some fidelity of the original organs. Organoids formed by the self-organization and differentiation of stem cells are a miniaturized organ model that preserves the fidelity of human organs 187. Taking the advantages of iPSCs, a personalized *in vitro* model can be established by growing organoids from patient-derived iPSCs. Besides, as each micrometre-size organoid can be served as one organ model, it is easier to accommodate multiple organoids in a single chip to realize higher-throughput screening. However, for the traditional 3D tissue models in bulk hydrogels, they have to be at least millimetre scale to acquire sufficient organ fidelity. The convergence of organoids and OOC platforms has been recently reported for the *in vitro* synovium model 182, 183. An OOC device containing a synovial organoid and a chondral organoid was developed to study the reciprocal crosstalk between synovium and cartilage with a higher degree of biomimicry in organ physiology and structure 188. Moreover, a synovial organoid-based OOC platform can be further incorporated with a light-scattering biosensor to allow the continuous monitoring of the disease progression on the chip 189. Therefore, we envision knee organoids (including cartilage organoids and bone organoids) to be the next-generation organ models for drug screening and disease modelling for OA. As for OA stem cell therapy, cartilage organoids or simply MSC spheroids have been reported to have a better regenerative capability than individual cells 182, 183. Delivering these multicellular spheroids or organoids to the knee joint could further improve the therapeutic efficacy of stem cell therapy, in particularly for the late-stage OA management. However, two major issues have to be addressed before knee organoids are widely used in OA research and treatment. On the one hand, it is vitally important to develop and standardize a cost-effective and robust protocol for growing organoids for a more popular application of organoids. On the other hand, specific microfluidic designs need to be developed to allow *i*) the growth of uniform organoids with precise control over the microenvironment for disease modelling and *ii*) the collection of these organoids and encapsulation of them to a proper vehicle for delivery.

### Multifunctional diagnosis from the synovial joint fluid

Although successful applications of microfluidics for the diagnosis of OA markers have been reported, microfluidics-based biosensors are still not the mainstream for OA diagnosis. This is because most of them requires an invasive collection of synovial joint fluid, which is undesired for early diagnosis. However, as synovial joint fluid is stored in the closed knee cavity, it is fundamentally impossible to conduct non-invasive OA diagnosis through microfluidics. Meanwhile, microfluidics is a powerful liquid biopsy tool that requires minimal samples but generates a significant amount of diagnostic information 190. Therefore, to expand the applications of microfluidics for OA diagnosis, developing multifunctional diagnosis from synovial joint fluid could be a direction. Since OA has been reported to be a risk factor or an early signal of many other age-related diseases including cardiovascular diseases 191 and diabetes 192, the markers present in the synovial joint fluid could contain more information just than the knee joint. Discovering the broader physiological significance of synovial joint fluid with microfluidics could improve the impact of microfluidics in diagnosis.

### Scaled-up device fabrication and automation operation

Even though microfluidics-based approaches have gained significant success on the benchtop, many of them are still away from being translated into clinical applications. One of the major obstacles is fabrication and operation. Conventional microfluidics is fabricated *via* photolithography of a silicon wafer master and soft lithography of PDMS devices. The fabrication of the master is time-consuming and cost-intensive while casting the PDMS devices from the master is labour-intensive and incompatible for automation. Therefore, plastic microfluidics or paper microfluidics could be a more suitable candidate for scaled-up fabrication. This is because they skip the need for photolithography of master and can be directly fabricated by machines such as micromilling 193 or printing 194 correspondingly in a fully automatic manner. In addition, the current microfluidic experiment requires skilful and well-trained personnel to operate the device. To replace human labour with a machine is highly desired in clinical applications to minimize human errors and increase the overall throughput. For example, incorporating a robotic liquid handler enables the automation in microfluidics operation 195.

Taken together, microfluidics is such a versatile and powerful tool that contributes to combating OA from all the different aspects including disease modelling, diagnosis, and therapy. Successful introduction of microfluidics to OA treatment brings a variety of novel and effective strategies to study and cure the disease. Having realized the importance of microfluidics to OA, it is expected that more and more microfluidic platforms will be developed to improve OA management.

## Figures and Tables

**Table 1:** Biomarkers of OA and their BIPED classifications

	Biomarker	Biological fluid	BIPED classification^78^
Cartilage degradation	MMP	Serum	B^79^ P^80^ E^81^ B^79^ P^80^ E^81^
	CTX-II	Urine, synovial fluid	B^82^ P^83^ E^81^ D^84^ B^82^ P^83^ E^81^ D^84^
	Coll2-1 NO2	Urine, serum	P^85^ P^85^
	C2C, C1, 2C	Urine, serum	P^86^ E^87^ P^86^ E^87^
Cartilage synthesis	CS846	Serum	E^88^ D^89^ E^88^ D^89^
	COMP	Serum	BP^90^ D^89^ BP^90^ D^89^
	YKL-40	Serum, synovial fluid	B^79^ E^91^ B^79^ E^91^
	PIIANP	Serum	P^92^ P^92^
	PIICP	Serum, synovial fluid	P^93^ D^94^ P^93^ D^94^
Bone degradation	NTX-I	Urine, serum	P^95^ D^96^ P^95^ D^96^
	CTX-I	Urine, serum	P^95^ P^95^
	D-PYR	Urine	B^79^ B^79^
Bone synthesis	OC	Serum	P^97^ P^97^
	BSP	Serum	D^98^ D^98^
Synovial degradation	Glc-Gal-PYR	Urine	D^99^ D^99^
	HA	Serum	B^79^ P^100^ B^79^ P^100^
Synovial Synthesis	PIIINP	Serum	P^101^ D^99^ P^101^ D^99^

BIPED classification: Burden of Disease, Investigative, Prognostic, Efficacy of Intervention and Diagnostic; BSP: bone sialoprotein; C2C, C1, 2C: collagenase-generated cleavage neoepitopes; Coll2-1 NO2: nitrated type II collagen degradation fragment; COMP: cartilage oligomeric matrix protein; CS846: chondroitin sulphate 846 epitopes; CTX-I: C-terminal telopeptide of collagen I; CTX-II: C-telopeptide of type II collagen fragment; D-PYR: deoxypyridinoline; Glc-Gal-PYR: glucosyl-galactosyl pyridinoline; HA: hyaluronic acid; MMP: matrix metalloproteinases; NTX-I: N-terminal telopeptide of collagen I; OC: osteocalcin; PIIANP: N-terminal propeptide of collagen IIA; PIICP: procollagen type II C-propeptide; PIIINP: type III procollagen; YKL-40: cartilage glycoprotein 39.

## Abbreviations

2D: two-dimensioinal
3D: three-dimensional
ADAMTS: thrombospondin type I motifs
ASCs: adipose-derived stem cells
BMSCs: bone marrow-derived stem cells
BSP: bone sialoprotein
C2C, C1, 2C: collagenase-generated cleavage neoepitopes
CM: chondrogenic differentiation medium
Coll2-1 NO2: nitrated type II collagen degradation fragment
COMP: cartilage oligomeric matrix protein
CS846: chondroitin sulphate 846 epitopes
CTX-I: C-terminal telopeptide of collagen I
CTX-II: C-telopeptide of type II collagen fragment
DEP: dielectrophoresis
D-PYR: deoxypyridinoline
ECM: extracellular matrix
ELISA: enzyme-linked immunosorbent assay
FACS: fluorescence-activated cell sorting
GAG: glycosaminoglycan
GelMA: gelatin methacryloyl
Gla: γ-carboxyglutamic
Glc-gal-PYR: glucosyl-galactosyl pyridinoline
HA: hyaluronic acid
hYKL-39: human cartilage chitinase-3-like protein 2
IL-8: interleukin-8
iPSCs: induced pluripotent stem cells
KS: keratan sulphate
MMP13: matrix metallopeptidase 13
MMPs: matrix metallopeptidases
MSCs: mesenchymal stem cells
NSAIDs: nonsteroidal anti-inflammatory drugs
NTX-I: N-terminal telopeptide of collagen I
OA: osteoarthritis
OC: osteocalcin
OM: osteogenic differentiation medium
OOC: organ-on-a-chip
PDMS: polydimethylsiloxane
PEG: poly(ethylene glycol)
PIIANP: N-terminal propeptide of collagen IIA
PIICP: procollagen type II C-propeptide
PIIINP: type III procollagen
PLGA: poly(lactic-co-glycolic) acid
PYR: pyridinoline
ROS: reactive oxygen species
SDF-1: stromal cell-derived factor-1
SIBLING: small integrin-binding ligand N-linked glycoproteins
YKL-40: cartilage glycoprotein 39
